# The prevalence of spontaneous pneumothorax in patients with BHD syndrome: a systematic review and meta-analysis

**DOI:** 10.1186/s13023-025-03726-z

**Published:** 2025-05-07

**Authors:** Yanan Zhang, Yuling Wang, Jinxia Wang, Ping Li, Ruonan Lv, Juan Chen

**Affiliations:** 1 Department of Respiratory and Critical Care Medicine, General Hospital of Ningxia Medical University, Yinchuan, 750004 Ningxia China; 2 Ningxia Medical University, Ningxia, 750004 China; 3 Department of Key Laboratory of Ningxia Stem Cell and Regenerative Medicine, Institute of Medical Sciences, General Hospital of Ningxia Medical University, Yinchuan, 750004 Ningxia China

**Keywords:** Birt-Hogg-Dubé syndrome, Spontaneous pneumothorax, Folliculin, Prevalence, Meta-analysis

## Abstract

**Background:**

Birt-Hogg-Dubé (BHD) syndrome is associated with an increased risk of pneumothorax. This study aimed to determine the prevalence of spontaneous pneumothorax among individuals diagnosed with BHD syndrome.

**Method:**

A comprehensive literature search was conducted across PubMed, EMBASE, Cochrane Controlled Register of Trials (CENTRAL), and Web of Science databases up to March 10, 2024. Studies reporting on the prevalence of spontaneous pneumothorax in BHD syndrome patients were included. Eligibility assessment, data extraction, and quality assessment were performed independently by two reviewers. Random-effects or fixed-effect models were conducted to calculate pooled incidence rates, and subgroup analyses were performed to explore sources of heterogeneity. The publication bias was assessed by funnel plot and Egger's test.

**Results:**

Eighteen studies, conducted between 2009 and 2023, were included in the systematic review. The meta-analysis revealed a pooled incidence rate of spontaneous pneumothorax in BHD syndrome patients at 0.61 (95% CI 0.46; 0.76). Subgroup analyses based on region, study design, and diagnostic methods further elucidated variations in incidence rates among different patient groups. Specifically, the Asian subgroup demonstrated a higher pooled incidence rate of 0.71 (95% CI 0.60; 0.81), while the Caucasian subgroup showed a lower pooled incidence rate of 0.43 (95% CI 0.26; 0.60). The subgroup analysis by study design revealed a pooled incidence rate of 0.60 (95% CI 0.45; 0.76) for retrospective studies and 0.70 (95% CI 0.42; 0.98) for the sole prospective study. Additionally, the subgroup analysis by diagnostic methods showed pooled incidence rates of 0.64 (95% CI 0.48; 0.81) for studies using *FLCN* mutation testing and 0.51 (95% CI 0.33; 0.70) for those using clinical criteria and imaging findings. Potential publication bias was identified by Egger's test (*P* < 0.05).

**Conclusion:**

The study indicated a pooled prevalence rate of 61% for pneumothorax in BHD syndrome patients, with subgroup analyses revealing higher rates among Asian individuals and in prospective studies. Further researches, particularly large-sample prospective studies, are needed to address publication bias and improve the reliability of prevalence estimates. PROSPERO: CRD42024567520.

**Supplementary Information:**

The online version contains supplementary material available at 10.1186/s13023-025-03726-z.

## Introduction

Birt-Hogg-Dubé (BHD) syndrome is a rare autosomal dominant genetic disorder, which was first described in 1977 by Birt, Hogg, and Dubé [1]. BHD syndrome is characterized by a spectrum of clinical manifestations affecting multiple organ systems, including cutaneous fibrofolliculomas, renal tumors, and an increased susceptibility to spontaneous pneumothorax [2, 3]. The underlying genetic defect responsible for BHD syndrome involves mutations in the folliculin (FLCN) gene located on chromosome 17p11.2 [4]. The primary diagnosis of BHD syndrome can be made using genetic testing, which detects specific mutations in FLCN [5]. The diagnosis can also be made based on clinical criteria including the presence of fibrofolliculomas, multiple lung cysts, renal tumors, and a relevant family history [2]. Among these manifestations of BHD syndrome, the recurrent occurrence of spontaneous pneumothorax is of particular concern due to its potential for life-threatening complications [6]. In Asian populations, 85–98% of patients with BHD syndrome exhibit pulmonary cystic changes, and despite most being asymptomatic, there is a significantly elevated risk of spontaneous pneumothorax, with 58–71% of these patients having a history of this condition [7].

Spontaneous pneumothorax refers to the sudden accumulation of air in the pleural cavity, the space between the lung and the chest wall, without any traumatic injury [8]. This condition can lead to lung collapse, resulting in symptoms such as sudden chest pain, shortness of breath, and difficulty breathing [9]. In severe cases, untreated pneumothorax can lead to significant dyspnea, thoracic pain, and decreased or absent breath sounds on auscultation. BHD syndrome presents with bilateral, multiple lung cysts that can rupture, leading to spontaneous pneumothorax, and patients with BHD are at high risk of recurrent pneumothorax [10]. Several studies have indicated the association of pneumothorax and BHD syndrome. Toro et al. conducted a study involving 198 patients with BHD syndrome, and revealed that pneumothorax occurring in approximately 24% of individuals [11]. Lee et al. reported a higher incidence of pneumothorax among individuals with BHD syndrome [12]. Specifically, in their study involving 12 patients diagnosed with BHD syndrome, 8 patients experienced pneumothorax [12]. However, despite several studies reporting on the incidence of pulmonary complications in individuals with BHD syndrome, the precise prevalence of these complications remained unclear. To address this, conducting a meta-analysis to calculate the pooled incidence rate is important. By synthesizing findings across various researches, meta-analysis enhanced the reliability and generalizability of the results, facilitating evidence-based decision-making in clinical practice. Moreover, gaining insights into the prevalence and clinical implications of spontaneous pneumothorax in BHD syndrome patients can help the development of targeted therapeutic interventions and preventive measures. Understanding how pulmonary involvement manifests in this population can guide clinicians in implementing personalized treatment plans and optimizing patient outcomes.

Therefore, the objective of this study was to systematically review the existing literature and perform a meta-analysis to analyze the prevalence of spontaneous pneumothorax among patients diagnosed with BHD syndrome.

## Methods

This systematic review and meta-analysis adhered to the Preferred Reporting Items for Systematic Reviews and Meta-Analyses (PRISMA) guidelines to evaluate prevalence of spontaneous pneumothorax in patients with BHD syndrome [13]. A systematic review protocol was developed and registered with International Prospective Register of Systematic Reviews (PROSPERO: CRD42024567520).

### Search strategy

A comprehensive literature search was conducted across four major databases from inception to March 10, 2024: PubMed, EMBASE, Cochrane Controlled Register of Trials (CENTRAL), and Web of Science, to identify studies reporting on the prevalence of spontaneous pneumothorax in patients with BHD syndrome. The search strategy was designed to encompass a wide range of terms related to BHD syndrome, and spontaneous pneumothorax, with the aim of capturing relevant studies without imposing restrictions on language or publication date. The detailed search strategy employed for each database was provided in Supplementary Table 1.

### Inclusion and exclusion criteria

The inclusion and exclusion criteria were as follows. The inclusion criteria included: (1) studies reporting patients with BHD syndrome; (2) studies reporting the incidence of spontaneous pneumothorax; (3) consecutive patients, defined as those included in the study based on the order in which they were diagnosed or presented. The exclusion criteria were as follows: (1) reviews; (2) conference papers; (3) case reports; (4) animal studies; (5) studies lacking specific data on patients diagnosed with BHD syndrome.

### Study selection

Two independent reviewers assessed the eligibility of retrieved records by screening their titles and abstracts. Subsequently, they thoroughly evaluated the full texts of potentially relevant studies. Any discrepancies were resolved through discussion or by consulting a third reviewer. The selection process was documented in a PRISMA flow diagram.

### Data extraction and quality assessment

Data extraction was performed by two independent reviewers based on the characteristics of the included studies. Each study was carefully reviewed to extract relevant information, including study design, sample size, patient demographics, diagnostic criteria for BHD syndrome, prevalence of spontaneous pneumothorax, pulmonary manifestations, and any additional outcomes of interest. The quality assessment of the included studies was conducted by two independent reviewers using the Methodological Index for Non-Randomized Studies (MINORS) score [14]. The MINORS score was a validated tool for assessing the methodological quality of non-randomized studies. It evaluates various aspects of study design, including the reporting of aims, patient selection, comparability of study groups, data collection methods, follow-up, and statistical analysis. Any discrepancies were resolved through discussion or consultation with a third reviewer.

### Data analysis

All statistical analyses were conducted using Stata 12.0 software. The prevalence of spontaneous pneumothorax in patients with BHD syndrome along with their corresponding 95% confidence intervals (CI) were calculated. The fixed-effects or random-effects models were adopted depending on the observed heterogeneity among the included studies. Heterogeneity was assessed using the I^2^ statistic, with values greater than 50% indicating substantial heterogeneity. For studies with low heterogeneity (I^2^ ≤ 50%), fixed-effects models were employed. Conversely, for those with high heterogeneity (I^2^ > 50%), random-effects models were utilized. Forest plots were generated to visually represent the prevalence and 95% CI of the included studies. Sensitivity analyses were performed using a leave-one-out approach, in which each study was individually removed from the meta-analysis to assess its impact on the overall results. Subgroup analyses were conducted to explore potential sources of heterogeneity and to examine the effects of various factors, including region, study design (prospective vs. retrospective), and diagnostic method (*FLCN* mutation testing vs. others) on the overall findings. Publication bias was assessed visually through funnel plot inspection and statistically using Egger's test [15]. In addition, to address publication bias, the trim-and-fill method was employed to estimate the number of potentially missing studies and adjust the effect size accordingly [16]. A significance level of *p* < 0.05 was considered statistically significant.

## Results

### Study selection

**Fig. 1:**
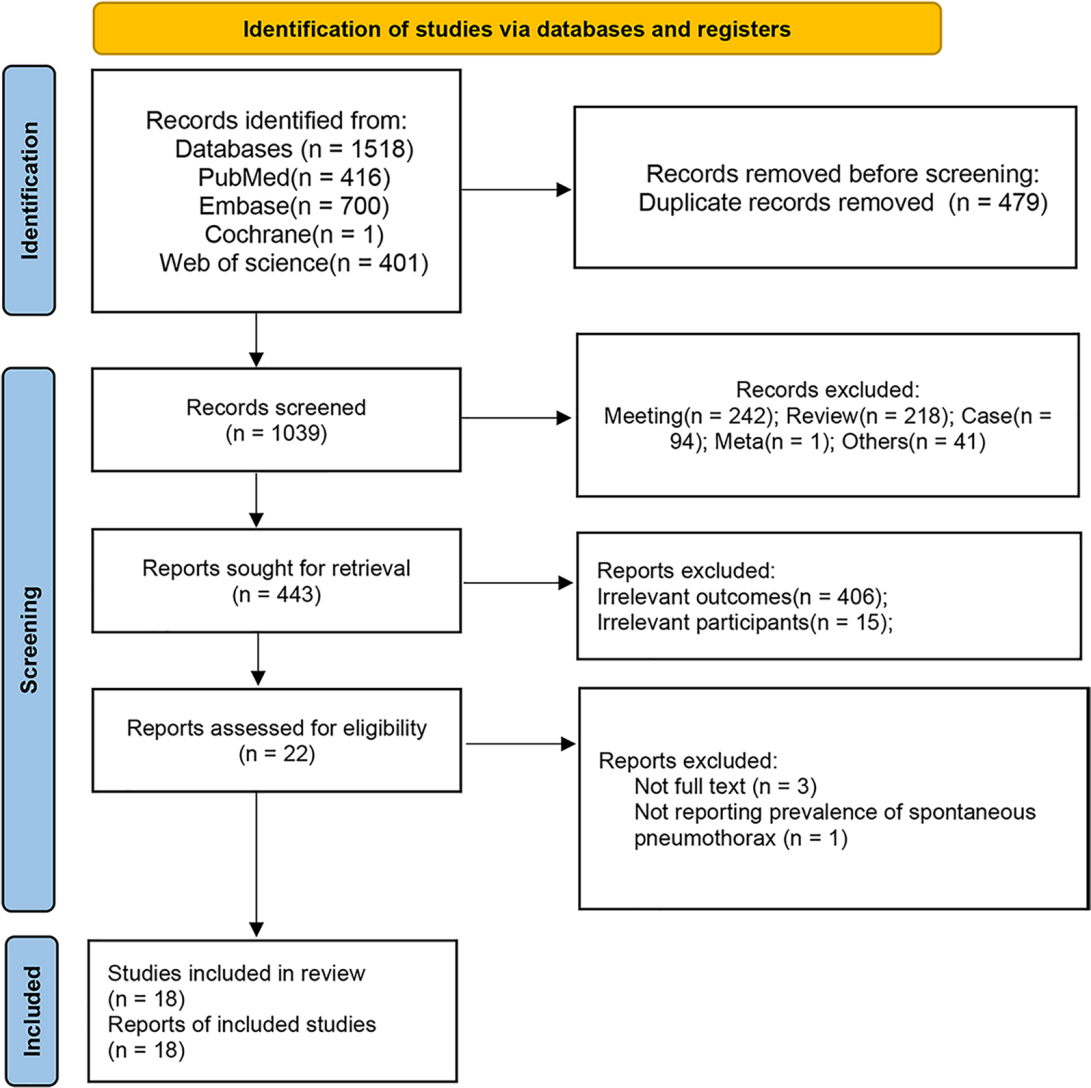
PRISMA study selection flow diagram. PRISMA, preferred reporting items for systematic reviews and meta-analyses

A comprehensive search across four databases including PubMed, Embase, Cochrane, and Web of Science was conducted, which resulted in the identification of 1518 records initially. After removing 479 duplicate records, 1039 records remained for screening on title and abstract. This screening stage excluded 596 records for various reasons: meeting abstracts (n = 242), review articles (n = 218), case reports (n = 94), meta-analysis (n = 1), and other irrelevant documents (n = 41), leaving 443 articles that were retrieved for further review. Of these, we excluded 421 reports for reasons including irrelevant outcomes and irrelevant participants. This process left 22 reports for a detailed eligibility assessment on full-text, with 3 records excluded due to unavailability of full text and one record excluded for not reporting spontaneous pneumothorax prevalence. Ultimately, 18 studies were included in the review (Fig. 1) [6, 11, 12, 17–31].

### Characteristics of included studies

This systematic review and meta-analysis included 18 studies, conducted between 2009 and 2023, which explored the genetic and clinical features of BHD syndrome across various countries including Korea, Germany, Japan, China, France, the Netherlands, and the United States (Table 1) [6, 11, 12, 17–31]. The majority of these studies were retrospective, apart from a single prospective study conducted in Korea in 2023 [26]. Diagnoses in these studies were mainly confirmed through *FLCN* mutation testing or a combination of clinical and imaging diagnostic criteria being the standard methods used to identify BHD syndrome. The total sample size across the studies varied, highlighting different scales of research with some studies involving as few as six participants and others including up to 334 participants. The radiological pulmonary manifestations were consistently documented, with some studies noting the progression in size and complexity of these cysts over time, providing valuable insights into the disease progression over time. Lung cysts were universally reported in BHD patients across all studies. The number and size of lung cysts varied significantly. Choi et al. observed that over 80% of patients exhibited more than 40 lung cysts, with a maximum diameter of approximately 4.1 cm [26]. Yang et al. documented 2323 lung cysts among 23 subjects, with sizes ranging from 4 to 110 mm [17]. Lung cyst morphology was diverse. Xu et al. reported the presence of fusiform cysts in 82% of patients, with a predominance near the mediastinum [18]. Additionally, Lee et al. [12] noted that cysts were predominantly found in the lower, peripheral, and subpleural regions of the lungs. Cho et al. [25] observed that the size of pulmonary cysts progressed over time in longitudinal follow-up thoracic CT in patients with BHD. Daccord et al. [32] found that 95% of BHD patients had multiple pulmonary cysts on CT.

**Table 1:** Characteristic of the included studies

Study	Study design	Country	Diagnostic methods	Sample sizes	age	Female %	Spontaneous pneumothorax	A family history of spontaneous pneumothorax	lung cyst	Renal lesions
[26]	Prospective study	Korea	FLCN mutation testing	10	49.4	80	7	30% patients had a family history of pneumothorax	80%	Cyst (50%)
[19]	Retrospective study	Germany	FLCN mutation testing	64	57.06	100	24	NR	NR	NR
[28]	Retrospective study	Japan	FLCN mutation testing	22	NR	50	15	NR	77.27%	Tumor (22.73%)
[23]	Retrospective study	Japan	FLCN mutation testing	30	48.76	80	29	63.3% patients had a family history of pneumothorax. 10% patients had a family history of skin lesion. 6.7% patients had a family history of renal disease	70%	Tumor (3.33%)
[12]	Retrospective study	Korea	FLCN mutation testing	12	41.3	66.7	8	41.67% patients had a family history of pneumothorax. 16.67% patients had a family history of skin lesion.8.3% patients had a family history of colon cancer	100%	Tumor (25%)
[20]	retrospective study	Korea	FLCN mutation testing	26	51	57.7	14	NR	NR	NR
[21]	Retrospective study	Japan	FLCN mutation testing	334	46	58.7	314	71.6% patients had a family history of pneumothorax. 16.1% patients had a family history of skin lesion.8% patients had a family history of renal tumors	100%	Tumor (3.7%); cyst (7.5%)
[17]	Retrospective study	China	FLCN mutation testing	26	46	61.54	14	NR	100%	Tumor (15.38%)
[25]	Retrospective study	Korea	the European BHD consortium	43	54.2	58.14	23	27.9% patients had a family history of pneumothorax	100%	NR
[22]	Retrospective study	China	FLCN mutation testing	27	48	88.89	20	81.48% patients had a family history of pneumothorax	92.59%	Tumor (7.4%); cyst (14.8%)
[32]	Retrospective study	France	The combination of clinical and imaging diagnostic criteria	96	48	48	57	61% patients had a family history of pneumothorax and/or pulmonary cysts	95%	Tumor (11%)
[18]	Retrospective study	China	FLCN mutation testing	33	48	93.9	17	75.8% patients had a family history of pneumothorax	100%	NR
[6]	Retrospective study	China	The criteria proposed by the European BHDS consortium	76	44	61.84	48	NR	93.42%	Tumor (5.26%)
[24]	Retrospective study	Netherlands	FLCN mutation testing	158	NR	NR	61	NR	100%	NR
[11]	Retrospective study	American	The presence of fibrofolliculom	198	49	48.99	48	15.66% patients had a family history of pneumothorax	89%	NR
[30]	Retrospective study	American	NR	104	47	82.69	79	NR	85%	Tumor (33%)
[29]	Retrospective study	China	the European BHD consortium	10	54.4	80	9	50% patients had a family history of pneumothorax	100%	Tumor (10%); cyst (20%)
[31]	Retrospective study	Netherlands	The presence of fibrofolliculom	115	NR	NR	28	NR	NR	Tumor (12%)

*NR* not reported

The quality of the included studies was assessed using the MINORS score (Table 2). A total of 14 studies scored 14 out of a possible 16 for non-comparative study criteria, reflecting a generally high level of methodological soundness. All of the four comparative studies demonstrated high methodological quality, which scored above 20. The quality assessment revealed a strong adherence to several critical methodological standards across the included studies.

**Table 2:** MINORS score for quality assessment of the included studies

Study/year	Methodological items												Total
	1	2	3	4	5	6	7	8	9	10	11	12	
[26]	2	2	2	2	2	2	2	2	2	2	2	2	24
[19]	2	2	2	2	2	2	2	0	2	2	2	2	22
[34]	2	2	2	2	2	2	2	0	0	0	0	0	14
[23]	2	2	2	2	2	2	2	0	2	2	2	2	22
[12]	2	2	2	2	2	2	2	0	0	0	0	0	14
[20]	2	2	2	2	2	2	2	0	0	0	0	0	14
[21]	2	2	2	2	2	2	2	0	0	0	0	0	14
[17]	2	2	2	2	2	2	2	0	0	0	0	0	14
[25]	2	2	2	2	2	2	2	0	0	0	0	0	14
[22]	2	2	2	2	2	2	2	0	0	0	0	0	14
[32]	2	2	2	2	2	2	2	0	0	0	0	0	14
[18]	2	2	2	2	2	2	2	0	0	0	0	0	14
[6]	2	2	2	2	2	2	2	0	2	2	2	2	22
[24]	2	2	2	2	2	2	2	0	0	0	0	0	14
[11]	2	2	2	2	2	2	2	0	0	0	0	0	14
[30]	2	2	2	2	2	2	2	0	0	0	0	0	14
[29]	2	2	2	2	2	2	2	0	0	0	0	0	14
[31]	2	2	2	2	2	2	2	0	0	0	0	0	14

The final score comprises the results of 8 items or 12 items in cases of comparative studies: 1 A clearly stated aim; 2 Inclusion of consecutive patients; 3 Prospective collection of data; 4 Endpoints appropriate to the aim of the study; 5 Unbiased evaluation of the study endpoint; 6 Follow-up period appropriate to the aim of the study; 7 Loss to follow-up less than 5%; 8 Prospective calculation of the study size; 9 An adequate control group; 10 Contemporary groups; 11 Baseline equivalence of groups; 12 Adequate statistical analysis

### Main results of the meta-analysis

**Fig. 2:**
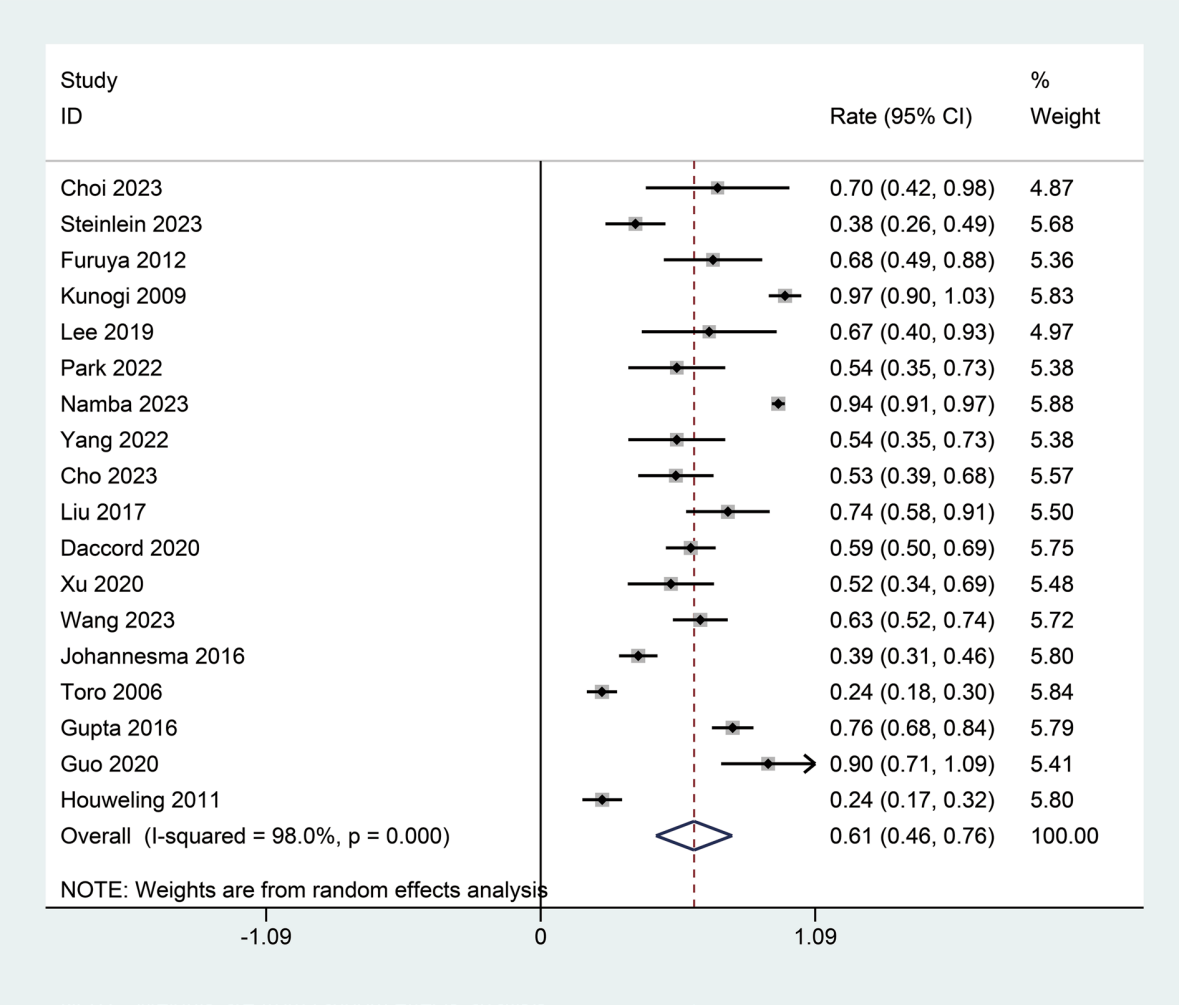
Forest plot of the incidence of spontaneous pneumothorax in patients with BHD syndrome

As illustrated in Fig. 2, a total of 18 studies reported the incidence of spontaneous pneumothorax in patients with BHD syndrome [6, 11, 12, 17–31]. This comprehensive analysis incorporated data from multiple studies, showing significant heterogeneity among the results (I^2^ = 98.0%, *p* < 0.01). Due to this observed heterogeneity, a random effects model was utilized to better account for the variance among the different studies. This model estimated a pooled incidence rate for spontaneous pneumothorax at 0.61 (95% CI 0.46; 0.76), indicating a relatively high incidence rate across the population studied. The sensitivity analysis confirmed that the results of the meta-analysis are stable and reliable (Supplemental Fig. 1). The minor fluctuations in the incidence estimates upon the exclusion of individual studies indicated that the overall conclusions drawn from the meta-analysis are robust against the potential bias of any single study (Supplemental Fig. 1).

### Subgroup analysis

The subgroup analysis of this meta-analysis investigated the variations in the incidence of spontaneous pneumothorax among patients with BHD syndrome based on region, study design, and diagnostic methods.

**Fig. 3:**
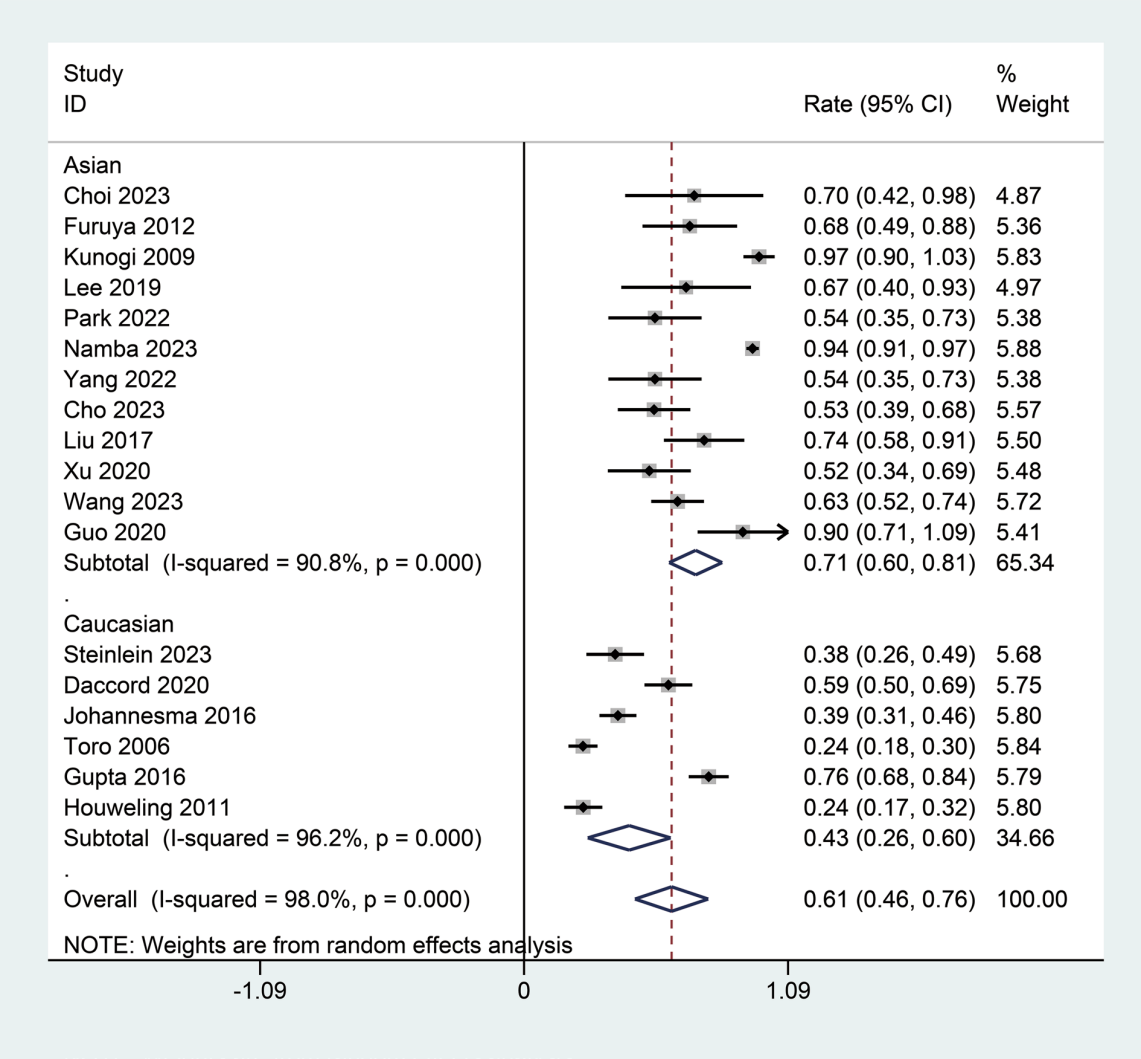
Subgroup analysis of the incidence of spontaneous pneumothorax in patients with BHD syndrome by region

The subgroup analysis by region divided studies into two groups: Asian and Caucasian. This Asian subgroup comprising 12 studies demonstrated a higher pooled incidence rate of 0.71 (95% CI 0.60; 0.81), with high heterogeneity (I^2^ = 90.8%, Fig. 3) [6, 12, 17, 18, 20–23, 25, 26, 28, 29]. This suggests that Asian patients may be more likely to develop or be diagnosed with spontaneous pneumothorax. In contrast, the Caucasian subgroup pooled six studies and showed a lower pooled incidence rate of 0.43 (95% CI 0.26; 0.60) with considerable heterogeneity (I^2^ = 96.2%, Fig. 3) [11, 19, 24, 27, 30, 31]. The differences between the Asian and Caucasian subgroups were statistically significant (*P* < 0.01).

**Fig. 4:**
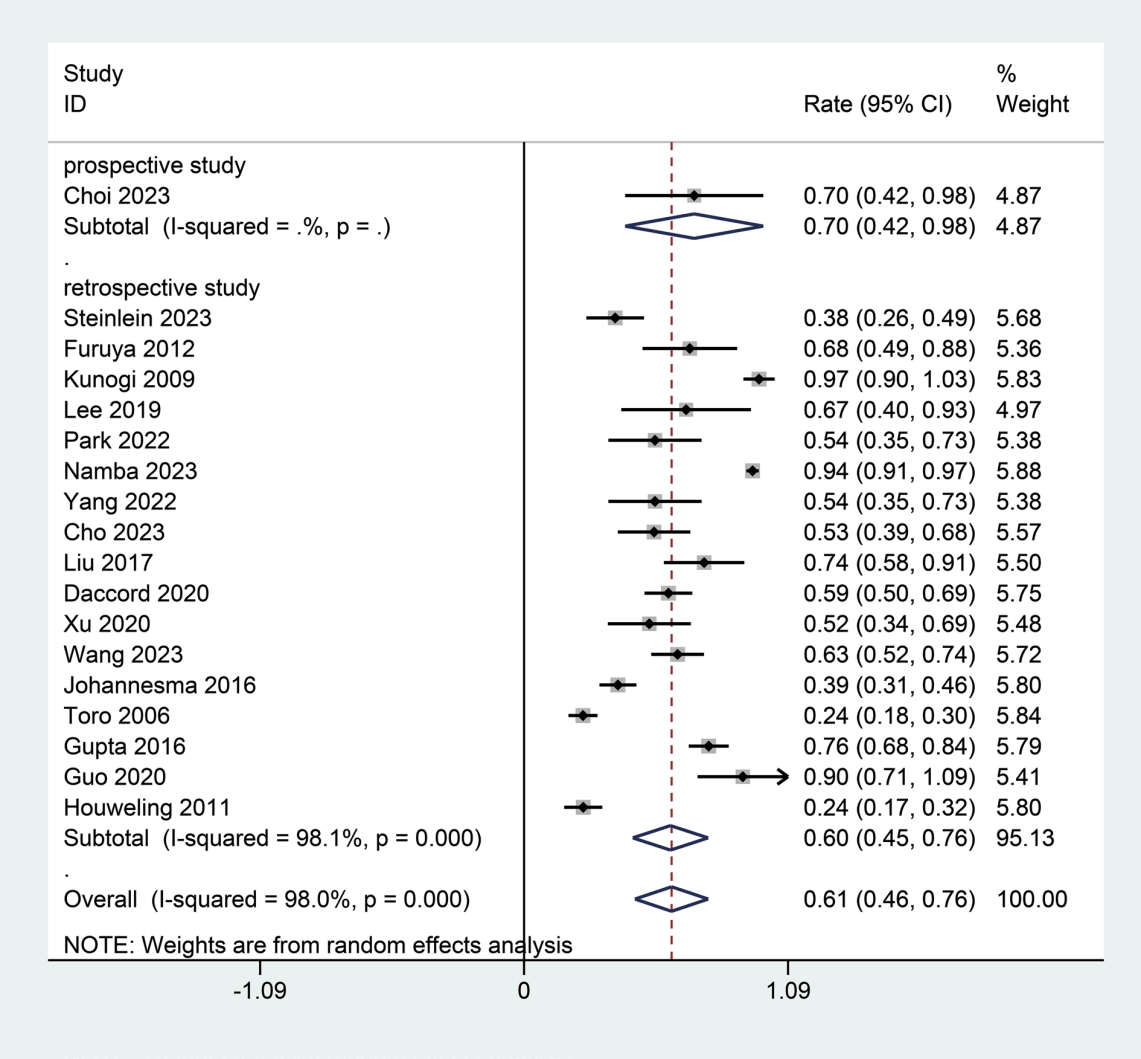
Subgroup analysis of the incidence of spontaneous pneumothorax in patients with BHD syndrome by study design

The subgroup analysis differentiating findings from retrospective and prospective studies provided the impact of study methodologies on reported incidence of spontaneous pneumothorax in patients with BHD syndrome. Retrospective studies subgroup included 17 studies [12, 19–25, 27–31]. The pooled incidence rate for retrospective studies was 0.60 (95% CI 0.45; 0.76), reflecting significant variability and a high degree of heterogeneity (I^2^ = 98.1%, Fig. 4). Choi et al. [26] conducted the sole prospective study, which reported a higher incidence rate of 0.70 (95% CI 0.42; 0.98) (Fig. 4). The differences between the retrospective and prospective subgroups were statistically significant (*P* < 0.01).

**Fig. 5:**
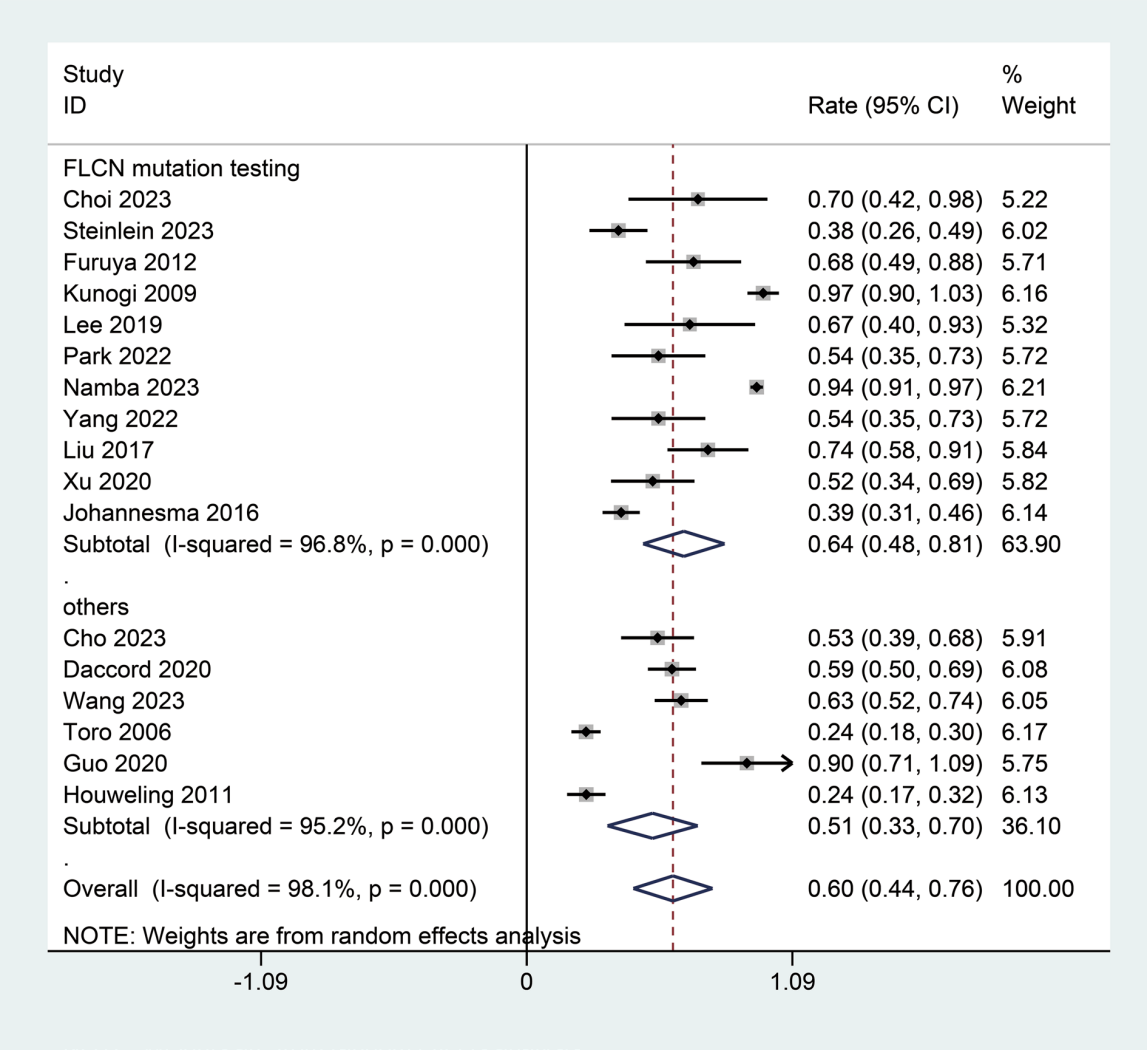
Subgroup analysis of the incidence of spontaneous pneumothorax in patients with BHD syndrome by diagnostic method

The subgroup analysis focusing on diagnostic methods specifically compared the incidence of spontaneous pneumothorax in patients with BHD syndrome based on whether *FLCN* mutation testing was used or other diagnostic criteria were applied. This subgroup using *FLCN* mutation testing reported high heterogeneity (I^2^ = 96.8%, Fig. 5) [12, 17–24, 26, 28]. The pooled incidence rate for this subgroup was 0.64 (95% CI 0.48; 0.81). Studies in subgroup that used clinical criteria and imaging findings to diagnosis BHD syndrome showed even higher heterogeneity (I^2^ = 95.2%, Fig. 5). The pooled incidence rate for this subgroup was 0.51 (95% CI 0.33; 0.70) (Fig. 5) [6, 11, 25, 27, 29, 31]. The differences between the subgroups were also statistically significant (*P* < 0.01).

### Publication bias

**Fig. 6:**
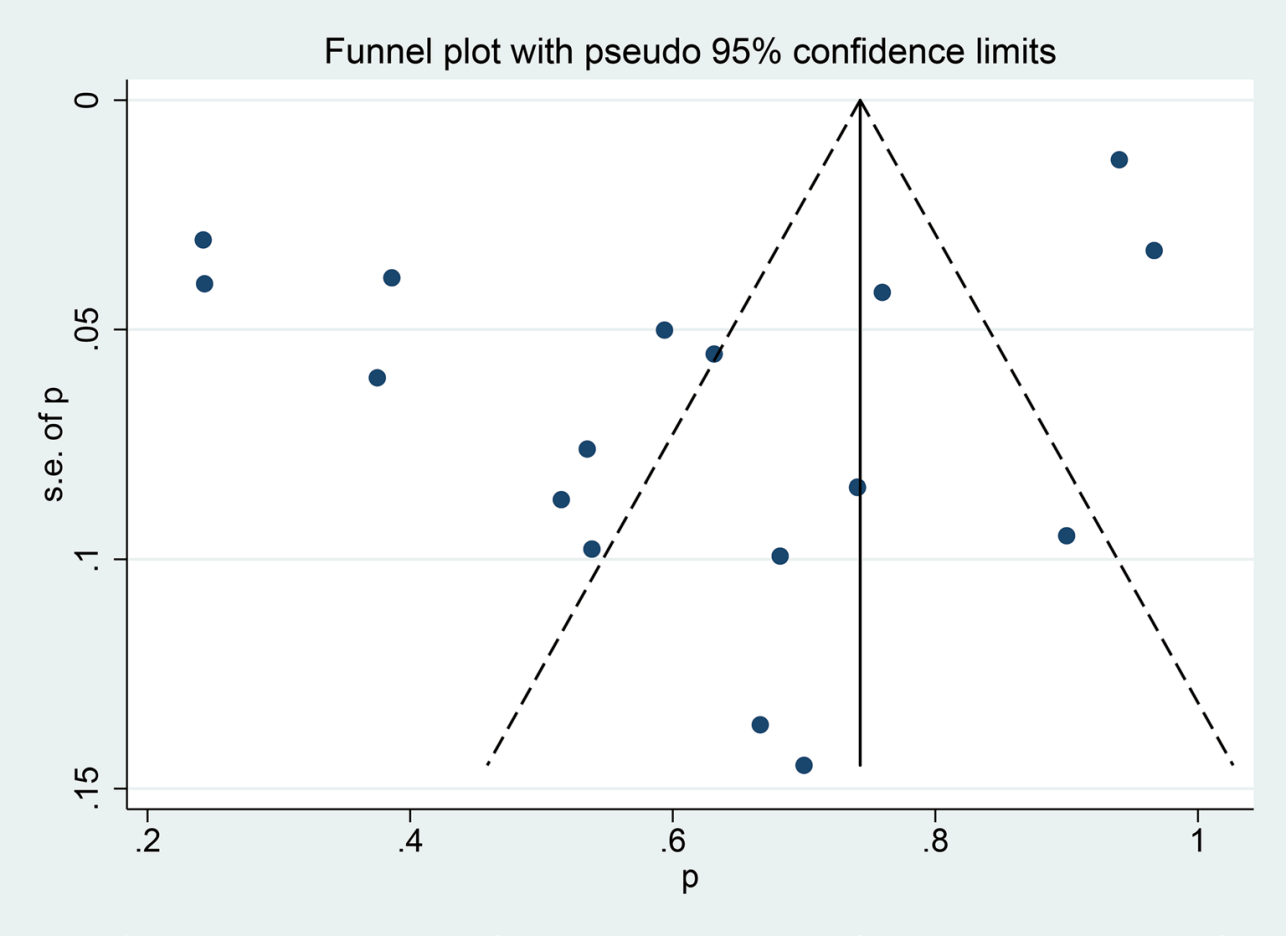
Funnel plot for publication bias

The funnel plot displayed an asymmetric distribution of dots around the pooled effect size, indicating potential publication bias (Fig. 6). Furthermore, Egger's test confirmed this bias with significant results (t = 9.34, *p* < 0.001), underscoring the presence of publication bias among the studies analyzed. The trimming procedure was adopted to investigate the effect of potential outliers or influential studies, which confirmed the robustness of the effect size estimate as stable.

## Discussion

This systematic review and meta-analysis included 18 studies to assess the prevalence of spontaneous pneumothorax in patients with BHD syndrome. Employing a random effects model to account for the observed heterogeneity across studies, this meta-analysis reported a pooled prevalence rate of spontaneous pneumothorax at 0.61 (95% CI 0.46; 0.76). This finding emphasized the significant clinical impact of BHD syndrome on pulmonary health. The subgroup revealed that Asian patients display a higher prevalence compared Caucasian patients. Moreover, prospective studies suggested higher prevalence rates compared with retrospective studies. Furthermore, studies utilizing *FLCN* mutation testing, a genetic marker for BHD syndrome, reported more consistent and slightly higher prevalence rates of pneumothorax compared to those employing other diagnostic criteria.

This meta-analysis uncovered a significant variation in prevalence rates across studies. Namba et al. [21] revealed that pneumothorax is prevalent in Asian patients with BHD syndrome, reporting an incidence rate of 94%. Houweling et al. [31] reported a high lifetime risk of pneumothorax risk (24%) in *FLCN* mutation carriers with BHD syndrome, which emphasized the importance of early surveillance and management strategies for BHD-associated conditions. Toro et al. [11] reported an incidence rate of 24% pneumothorax in patients with BHD syndrome, and indicated a significant association between lung cysts and spontaneous pneumothorax. Such variability underscored the necessity for sensitivity analysis to confirm the stability and reliability of our meta-analysis findings. The sensitivity analysis reported that excluded additional studies showed only minor variations in the pooled estimates, which highlighted the overall robustness of our meta-analysis findings. The asymmetric funnel plot and Egger's test suggested potential publication bias in the included studies. To address this, we employed the trim and fill method, a recognized technique for evaluating and adjusting for publication bias in meta-analyses [16]. This method further confirmed the robustness of the result.

The subgroup analysis indicated a notable difference in prevalence between Asian individuals and Caucasian individuals, with Asian individuals showing a higher prevalence. These differences are likely influenced by genetic and ethnic factors that affect the clinical manifestations of the syndrome. Sattler et al. found that in Caucasian patients with BHD syndrome, significant differences in pneumothorax risk were observed based on age, sex, and specific FLCN mutations, with the highest risks associated with mutations c.1300G > C and c.250-2A > G[33]. Specific *FLCN* mutations found in Asian populations might predispose these individuals to a higher risk of developing pulmonary cysts and spontaneous pneumothorax [34]. Currently, there are no studies comparing the frequencies of the mutations identified in Asian and Caucasian populations with BHD syndrome. Further research is needed to conduct a comparative analysis of these mutations across different ethnic groups to better understand the potential genetic and epidemiological differences. The observed regional differences in the incidence of spontaneous pneumothorax have important implications for clinical practice and patient management, and clinicians should be aware of these disparities and consider regional and ethnic factors when diagnosing and treating BHD syndrome. Previous study identified that the number of cysts located on the pleural surface, along with cyst size, cyst number, and cyst volume, are key factors associated with an increased risk of spontaneous pneumothorax in patients with BHD syndrome [11]. The subgroup analysis suggested that prospective studies report higher prevalence rates than retrospective studies, which suggested potential methodological differences that may influence the reported incidence of pneumothorax risk in patients with BHD syndrome. Prospective studies typically involve more rigorous data collection protocols, including regular follow-up examinations and standardized diagnostic criteria, which may enhance the detection of pneumothorax cases compared to retrospective studies [26]. Additionally, prospective studies may have longer study durations, allowing for a more comprehensive assessment of pneumothorax occurrence over time. However, only one study included in the analysis was prospective, highlighting the need for more prospective large-scale studies to validate and further elucidate the prevalence rates of pneumothorax risk in patients with BHD syndrome [26]. Previous studies have suggested that BHD syndrome is caused by mutations in the folliculin (*FLCN*) gene located on chromosome 17p11.2 [4, 35]. The *FLCN* gene encodes the folliculin protein, which plays a role in cell signaling pathways and cellular metabolism regulation [36]. *FLCN* mutation testing served as a genetic marker for BHD syndrome and enabled more precise identification of affected individuals [2]. Mutations in the *FLCN* gene cause functional loss of the folliculin protein, and these mutations can occur at various positions within the gene. Patients with *FLCN* mutations in exons 9 and 12 had a higher frequency of pneumothorax compared to patients with mutations in other exons [11]. Studies using *FLCN* mutation testing may thus capture a more homogeneous population with a higher likelihood of having BHD syndrome, leading to more consistent and slightly higher prevalence rates of pneumothorax. Furthermore, Wang et al. reported that the deletion of exons 1–3 in *FLCN* was associated with a significantly higher risk of pneumothorax compared to those with point mutations, underscoring the complex relationship between *FLCN* mutations and clinical outcomes in BHD syndrome [6].

This study has several limitations to be addressed. First, only one prospective study was included in this study, and the lack of sufficient prospective data might restrict the ability to draw firm conclusions about the prevalence of spontaneous pneumothorax in patients with BHD syndrome. More prospective cohort studies should be conducted to provide high-quality evidence. Second, the Egger's test indicated the presence of publication bias, leading to an overestimation of the true effect size. Further large-sample prospective studies should be conducted to offer advantages in addressing publication bias and improving the validity of research outcomes. Additionally, several studies included in the analysis originated from the same hospital, which could introduce potential bias. Future research should include data from a wider range of institutions to improve the generalizability of the results.

### Conclusion

In conclusion, this systematic review and meta-analysis assessed the prevalence of spontaneous pneumothorax in patients with BHD syndrome, revealing a pooled prevalence rate at 61%. Subgroup analyses highlighted higher prevalence rates of pneumothorax among Asian individual and a tendency for prospective studies to report higher rates. Furthermore, the presence of publication bias underscored the necessity for large-sample prospective studies to enhance the reliability of the results.

## Supplementary Information

Additional file1

## Data Availability

All data generated or analysed during this study are included in this published article.
