# Supplementary material for: The prevalence of spontaneous pneumothorax in patients with BHD syndrome: a systematic review and meta-analysis
Source: Orphanet J Rare Dis. 2025 May 7;20:218. doi: 10.1186/s13023-025-03726-z (PMC12060348; doi:10.1186/s13023-025-03726-z)

**Supplemental Figure 1. Sensitivity Analysis of Incidence of Spontaneous Pneumothorax in Patients with BHD syndrome**
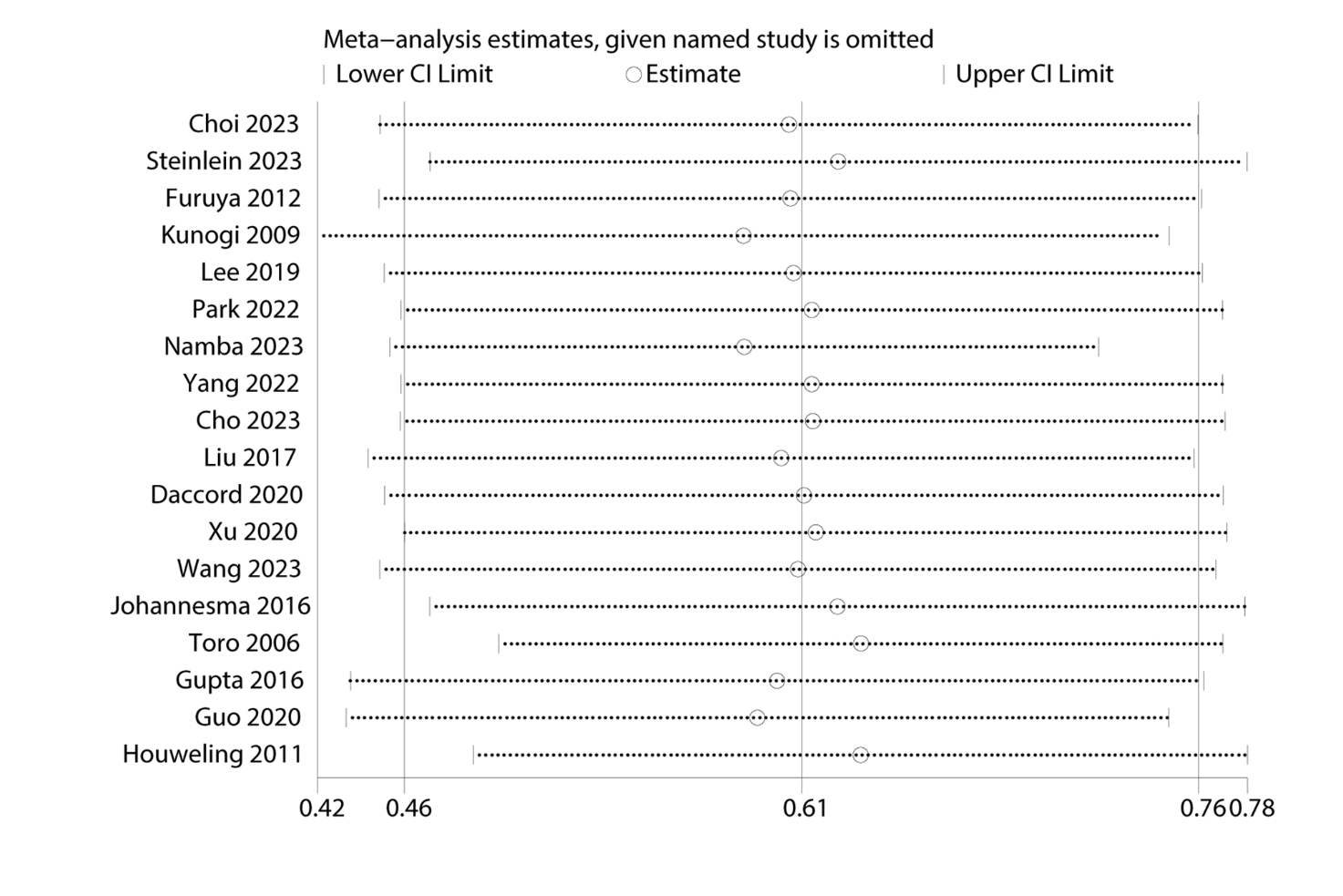

Supplement: Supplementary file 1 — Additional file1 [file 13023_2025_3726_MOESM1_ESM.docx]
